# A cross-sectional study on fear of progression in patients with myasthenia gravis

**DOI:** 10.1038/s41598-025-11806-0

**Published:** 2025-07-18

**Authors:** Si Luo, Ying Xiong, Ziwei Song, Jia Lian, Yusen Qiu, Min Zhu, Menghua Li, Meihong Zhou, Daojun Hong

**Affiliations:** 1 Department of Neurology, The First Affiliated Hospital, Jiangxi Medical College, Nanchang University, Nanchang, China; 2 Rare Disease Center, The First Affiliated Hospital, Jiangxi Medical College, Nanchang University, Nanchang, China; 3 Institute of Neurology, Jiangxi Academy of Clinical Medical Science, The First Affiliated Hospital, Jiangxi Medical College, Nanchang University, Nanchang, China

**Keywords:** Fear of progression, Myasthenia gravis, Gender, Progress, Health care, Neurological disorders

## Abstract

Patients with myasthenia gravis (MG) are prone to resulting in a significant psychological burden. This study aims to investigate the condition of fear of progression (FoP) and identify factors associated with FoP among MG patients. This cross-sectional study included 83 patients with MG admitted to the First Affiliated Hospital of Nanchang University from January to November 2024. Data were collected through clinical records and questionnaires, including demographic details, disease duration, comorbidities, current drug regimen, and the fear of progression questionnaire-short form (FoP-Q-SF). The simplified fear of progression scale score for 83 MG patients was 31.36 ± 10.22. Among these participants, 38.6% exhibited dysfunction FoP. There was no significant difference concerning age, marital status, course of the disease, crisis of MG, STAI score, and TAI score in the total score of fear of disease progression in MG patients. However, gender, progress of MG and MGFA classification were positively correlated with the fear of progression (*P* < 0.05), which was statistically significant. Furthermore, gender and disease progression were identified as factors influencing FoP in MG patients (*P* < 0.05). FoP among MG patients varies according to their socio-demographic and clinical characteristics. Gender and progress history of MG are associated with Fop.

**Supplementary Information:**

The online version contains supplementary material available at 10.1038/s41598-025-11806-0.

## Introduction

Myasthenia gravis (MG) is an autoimmune disease in which antibodies bind to acetylcholine receptors or molecules located on the postsynaptic membrane at the neuromuscular junction. The estimated prevalence of MG is approximately 20 individuals per 100,000 persons^1^. Weakness is the most common symptom and other clinical symptoms include double vision, drooping eyelids, difficulties with speech and swallowing, respiratory muscle weakness, and fatigue in specific muscles, and the weakness increases with exercise and fatigue^2^. The severity of the disease varies considerably between individuals, with its course characterized by relapses and occasional periods of remission^3^. Beyond the weakness symptoms, MG patients may also experience psychological distress due to complications and recurrent exacerbations^4^. Previous studies indicate that mood and anxiety disorders are the most common psychological outcomes among MG patients^5–7^. Furthermore, MG patients encounter different and complex treatment regimens that may lead to adverse drug effects and negatively impact their quality of life^8^. As a result, patients often experience heightened concerns about disease progression or recurrence, resulting in a significant psychological burden.

Fear of progression (FoP) refers to the patients’ fear of biological or psychological consequences due to the development or recurrence of their diseases^9–11^. It is prevalent among patients with cancer and those with chronic diseases. FoP can be considered a normal psychological response, which may even serve adaptive functions under certain circumstances^12^. However, when FoP elevates to a dysfunctional degree, such as potentially interfering with treatment adherence or daily functioning, interventions become necessary. Previous research indicates that FoP is one of the significant emotional difficulties faced by patients with chronic progressive diseases^9^. Additionally,

FoP can be perceived as a threatening experience that threatens the life of patients^11–13^, affecting their behavioral dysfunction, well-being, mental health, and quality of life^11,14,15^. Currently, there is no established data on FoP among MG patients. The objective of this study is to investigate the current state of FoP and identify factors associated with FoP in MG patients. Identifying these factors may assist us in identifying high-risk patients and developing effective intervention strategies to alleviate FoP in MG patients.

## Materials and methods

### Study design

This study is a cross-sectional study aimed to investigate the current situation of Fop among MG patients and analyze its associated factors. The study was conducted at the First Affiliated Hospital of Nanchang University between January 2024 and November 2024. Informed consent was obtained from all participants before they participated in the research. Enrolled patients met the following criteria: (1) typical clinical manifestations of MG; (2) at least one positive result from neostigmine testing, repetitive nerve stimulation (RNS), or acetylcholine receptor (AChR) antibody testing; and (3) exclusion of alternative diagnoses prior to MG confirmation; (4) ability to understand and complete the questionnaire voluntarily. Patients aged younger than 14 years at the time of disease onset were excluded from this study.

### Data collection

Data collection consisted of two principal components: clinical data and questionnaire responses. Clinical data were collected by attending physicians based on patient medical records and physical examination findings. This information included demographic information including age, gender, disease duration, comorbidities, and drug regimen. Specifically, the Myasthenia Gravis Foundation of America (MGFA) classification and Myasthenia Gravis Activities of Daily Living (ADL) score were used to assess disease severity. The scale of the Spielberger State-Trait Anxiety Inventory (STAI) which consisted of the State Anxiety Inventory (SAI) and Trait Anxiety Inventory (TAI) was applied to evaluate the level of anxiety. The scores of each subscale could range from a minimum of 20 to a maximum of 80. A score of 20–31 reflected mild anxiety, 32–42 below moderate anxiety, 43–53 above moderate anxiety, 54–64 relatively severe anxiety, and 65–75 severe anxiety^16^. A cut-off of 43 or higher was considered indicative of dysfunctional anxiety. Questionnaire data were collected in a face-to-face manner, and it was completed by the patients independently. For those unable to do so, the researchers asked questions according to the contents of the questionnaire and assisted them in completing it.

### Fear of progression questionnaire

Participants completed the Fear of Progression Short Form (FoP-Q-SF), developed by Mehnert et al. based on the Fear of Progression Questionnaire (FoP-Q)^17^. This scale comprises two dimensions: social family and physiological health, with a total of 12 items rated on a five-point scale ranging from “never” to “very often”. Total scores ranged from 12 to 60, with higher scores indicating greater levels of fear, with a cut-off of 34 or above indicating a dysfunctional level of fear of progression^18^.

### Statistical analysis

All statistical analyses were conducted using SPSS version 26.0 statistical software. Descriptive statistics were used for demographic and disease-related data. Counting data were described as frequency and percentage. In single-factor analysis, independent sample t-tests or single-factor ANOVAs were employed. Non-parametric tests were used to analyze how various demographic factors and disease-related variables impacted FoP levels in MG patients. Spearman’s Rank Correlation Analysis was applied to assess relationships between FoP levels and MG-ADL scores, MGFA classifications, STAI scores, and TAI scores. Variables with *P* < 0.05 in single-factor analysis were included in the multiple linear regression model to identify influencing factors of FoP.

### Ethical approval and patient consent

This study was approved by the Ethics Committee of First Affiliated Hospital of Nanchang University with approval number AF-SG-03-2.1-IIT. The patients provided written informed consent to their participation in the study and were authorized to publish the study. All research was performed in accordance with relevant guidelines.

## Results

### Characteristics of the participants

Among the 83 included MG patients, 100% (*n* = 83) tested positive for AChR antibodies. RNS was performed in 39 patients (46.99%), of whom 22 (56.41%) showed abnormalities suggestive of MG. The neostigmine test was performed in 68 patients (81.93%), with 33 (48.53%) demonstrating a positive response. Detailed diagnostic test results are presented in (Table S1). The demographic characteristics of the 83 enrolled MG patients are shown in Table 1. The cohort included 42 males (50.6%) and 41 females (49.4%), with a mean age of 45.18 ± 17.92 years. Most patients were between 40 and 65 years old (44.6%), followed by 27.7% aged 18–40. Regarding marital status, 69.9% were married, while 19.3% were single. In terms of employment, 41% were freelancers, 31.3% employed, 15.7% unemployed, and 12% retired. Monthly income varied, with 30.1% earning below 3000 yuan, and only 10.8% earning more than 10,000 yuan. Educational background was relatively low, with 21.7% having primary education or below, and only 8.4% having a bachelor’s degree or higher. The clinical characteristics of the 83 MG patients are summarized in Table 2. Most patients were classified as MGFA Class I (71.1%), followed by Class II (22.9%). The majority had a disease duration between 1 and 5 years (39.8%), with 30.1% newly diagnosed (< 1 year) and 19.3% with disease duration over 10 years. Disease progression was reported in 37.3% of patients, while MG crisis was rare, occurring in only 3.6% of the cohort. Nearly half of the patients (48.2%) had at least one chronic comorbidity, with the most common types including hypertension, diabetes, and thyroid dysfunction (Table S2). Thymoma was present in 9.6% of the patients. Regarding treatment, 61.4% of patients were receiving corticosteroids, and an equal proportion (61.4%) were treated with immunosuppressants.

**Table 1:** Demographic data of patients with myasthenia gravis.

Variables	*n*	Percentage(%)
**Gender**		
Male	42	50.6
Female	41	49.4
**Age(year)**		
≥ 14 ≤ 18	9	10.8
>18 ≤ 40	23	27.7
≥ 40<65	37	44.6
≥ 65	14	16.9
**Marital status**		
Single	16	19.3
Married	58	69.9
Divorced	3	3.6
Widowed	6	7.2
**Employment**		
Employed	26	31.3
Unemployed	13	15.7
Retired	10	12.0
Freelancer	34	41.0
**Monthly income(yuan)**		
>3000	25	30.1
≥ 3000<5000	26	31.3
≥ 5000<10,000	23	27.7
≥ 10,000	9	10.8
**Education**		
Primary school or below	18	21.7
Junior high school	25	30.1
Senior high school	16	19.3
Associate degree	16	19.3
Bachelor’s degree or higher	7	8.4

**Table 2:** Disease-related data of patients with myasthenia gravis.

Variables	*n*	Percentage(%)
**MGFA**		
Class I	59	71.1
Class II	19	22.9
Class III	2	2.4
Class IV	1	1.2
Class V	2	2.4
**MGFA I subtypes**		
Pure ocular MG	55	66.3
Residual ocular symptoms after generalization	4	4.8
**MG subtypes**		
OMG	59	71.1
EOMG	13	15.7
LOMG	9	10.8
TAMG	2	2.4
**Course of disease(year)**		
<1	25	30.1
≥ 1<5	33	39.8
≥ 5<10	9	10.8
≥ 10	16	19.3
**Progress of MG**		
Yes	31	37.3
No	52	62.7
**Crisis of MG**		
Yes	3	3.6
No	80	96.4
**Chronic comorbidities**		
Yes	40	48.2
No	43	51.8
**Thymoma**		
Yes	8	9.6
No	75	90.4
**Corticosteroids**		
Yes	51	61.4
No	32	38.6
**Immunosuppressant**		
Yes	51	61.4
No	32	38.6

### Fear of progression in MG patients

In this study, the mean total score of Fop among 83 MG patients was 31.36 ± 10.22. The multiple dimensions of disease progression-related fear include physical health as well as social and familial aspects, with mean scores of fear being 15.87 ± 5.27 and 15.49 ± 5.63 respectively. These results suggest that MG patients experience comparable psychological stress both regarding physical health concerns and the impact of illness on social and family life. Details are summarized in Table 3. Among the items of the simplified Fear of Progression Questionnaire-Short Form, item 10 “I’m afraid the drugs will damage my body” had the highest score of 3.14 ± 1.01, followed closely by item 4 “The thought of being less productive because of illness annoys me” at 2.96 ± 1.19 and item 12 “The idea that I might not be able to work because of illness bothers me” at 2.89 ± 1.20 (Table 4). Furthermore, based on the established cut-off score of 34 for dysfunctional fear of progression, 32 patients (38.6%) were classified as having dysfunctional FoP, while 51 patients (61.4%) had non-dysfunctional levels. This indicates that more than one-third of MG patients in this cohort experienced a clinically relevant level of fear that may interfere with daily functioning or psychological well-being (Table 5).

**Table 3:** Fear of progression scores in patients with myasthenia gravis.

Variables	Scoring range	Minimum	Maximum	Score
Fear in the socio-family dimension	1—5	12	60	15.49 ± 5.63
Fear in the Physiological Health Dimension	1—5	6	30	15.87 ± 5.27
Fear of progression	12—60	6	30	31.36 ± 10.22

**Table 4:** Scores of each item of the fear of progression scale in patients with myasthenia gravis.

Item No.	Item Content	Score
1	I became anxious at the thought of the disease progressing.	2.80 ± 1.09
2	I feel nervous before a doctor’s exam and some regular medical check-ups.	2.30 ± 1.17
3	I’m afraid of the pain caused by this disease.	2.49 ± 1.13
4	The thought of being less productive because of illness annoys me.	2.96 ± 1.19
5	I have some physical discomfort when I am anxious (e.g., rapid heartbeat, stomach pain, nervousness, etc.).	2.31 ± 1.14
6	I’m worried that my disease might be passed on to my children.	2.07 ± 1.21
7	The possibility of having to rely on strangers in my daily life makes me anxious.	2.06 ± 1.23
8	I worry that I will not be able to continue my hobbies/hobbies at some point due to illness.	2.65 ± 1.24
9	I fear that there will be some major treatment in the course of the disease.	2.82 ± 1.00
10	I’m afraid the drugs will damage my body.	3.14 ± 1.01
11	I worry about what will happen to my family if something happens to me.	2.86 ± 1.26
12	The idea that I might not be able to work because of illness bothers me.	2.89 ± 1.20

**Table 5:** Dysfunctions of fear of progression in patients with myasthenia gravis.

Degree of fear of progression	*n*	Percentage(%)
The fear of progression is dysfunctional (FoP score ≥ 34)	32	38.6
The fear of progression is not dysfunctional (FoP score < 34)	51	61.4

### Influencing factors of fear of progression in patients with MG

The results revealed that age (*P* = 0.33), marital status (*P* = 0.53), disease course (*P* = 0.99) and crisis of MG (*P* = 0.87) demonstrated no statistically significant differences in total FoP scores. Comparatively, MG patients aged ≤ 18 years exhibited a total FoP score of 32.67 ± 9.77, followed by 33.52 ± 9.59 for those aged > 18 to 40 years, and 29.05 ± 9.19 for individuals ≥ 40 and < 65 years. Among elderly patients (≥ 65 years), the score was 33.07 ± 13.53. Although not statistically significant, MG patients with a history of disease crisis exhibited a slightly higher total FoP score at 32.33 ± 2.08 compared to those without at 31.32 ± 10.40 (Table 6). Among the 59 patients classified as MGFA Class I, 55 patients were identified as having pure ocular myasthenia gravis from onset, while 4 patients had previously presented with generalized symptoms but retained only ocular symptoms at the time of evaluation. Subgroup analysis revealed that patients with pure ocular MG had lower FoP scores at 29.81 ± 10.72 compared to those with residual ocular symptoms following generalized MG at 30.75 ± 11.81. Although this difference was not statistically significant, it suggests that disease phenotype may influence patients’ psychological perception of disease progression.

**Table 6:** Fear of disease progression by demographic profile.

Variables	Total fear of progression(Mean ± SD)	t/F-value	*P*
**Gender**			
Male(*n* = 42)	28.07 ± 9.94	−3.12	0.002*
Female(*n* = 41)	34.73 ± 9.48		
**Age(years)**			
≥ 14 ≤ 18(*n* = 9)	32.67 ± 9.77	1.15	0.33
>18 ≤ 40(*n* = 23)	33.52 ± 9.59		
≥ 40<65(*n* = 37)	29.05 ± 9.19		
≥ 65(*n* = 14)	33.07 ± 13.53		
**Marital status**			
Single(*n* = 16)	31.81 ± 9.55	0.75	0.53
Married(*n* = 58)	30.62 ± 10.23		
Divorced(*n* = 3)	39.00 ± 9.64		
Widowed(*n* = 6)	33.50 ± 12.76		
**Employment**			
Employed	30.85 ± 8.28	1.79	0.16
Unemployed	27.46 ± 9.85		
Retired	37.20 ± 13.45		
Freelancer	31.53 ± 10.32		
**Monthly income(yuan)**			
<3000(*n* = 25)	33.32 ± 11.07	1.11	0.35
≥ 3000<5000(*n* = 26)	31.77 ± 10.12		
≥ 5000<10,000(*n* = 23)	30.78 ± 9.35		
≥ 10,000(*n* = 9)	26.22 ± 10.02		
**Education**			
Primary school or below	33.72 ± 12.23	1.36	0.25
Junior high school	30.40 ± 11.06		
Senior high school	28.25 ± 8.93		
Associate degree	33.00 ± 7.44		
Bachelor’s degree or higher	35.57 ± 7.89		
**The course of the disease(years)**			
<1(*n* = 25)	31.72 ± 10.90	0.35	0.99
≥ 1<5(*n* = 33)	31.18 ± 11.14		
≥ 5<10(*n* = 9)	30.56 ± 8.90		
≥ 10(*n* = 16)	31.63 ± 8.57		
**Progress of MG**			
Yes	36.00 ± 7.75	3.39	0.001*
No	28.60 ± 10.58		
**Crisis of MG**			
Yes	32.33 ± 2.08	0.17	0.87
No	31.32 ± 10.40		
**Chronic comorbidities**			
Yes	30.83 ± 11.61	−0.46	0.65
No	31.86 ± 8.85		
**Thymoma**			
Yes	29.13 ± 8.80	−0.65	0.52
No	31.60 ± 10.39		
**Corticosteroids**			
Yes	31.43 ± 9.94	0.08	0.94
No	31.25 ± 10.81		
**Immunosuppressant**			
Yes	32.06 ± 9.45	0.78	0.44
No	30.25 ± 11.42		
**MGFA I subtypes**			
Pure ocular MG	29.81 ± 10.72	−0.17	0.87
Residual ocular symptoms after generalization	30.75 ± 11.81		
**MG subtypes**			
OMG	29.88 ± 10.68	1.59	0.20
EOMG	35.07 ± 4.55		
LOMG	34.00 ± 12.24		
TAMG	39.00 ± 2.83		

Abbreviations: *p < 0.05.

On the other contrary, there were significant differences in the total FoP scores between genders and the progress of MG in patients of this study, with P-values < 0.05. Specifically, the FoP score was significantly higher among female patients at 34.73 ± 9.48 compared to their male counterparts at 28.07 ± 9.94. Additionally, MG patients with a history of disease progression exhibited a notably higher FoP score at 36.00 ± 7.75 compared to those without such a history at 28.60 ± 10.58. Spearman’s Rank Correlation analysis further revealed that there were no statistically significant differences in MG-ADL scores, STAI scores, or TAI scores among MG patients. However, there was a positive correlation existed between the FoP score and the classic MGFA classification (*r* = 0.229, *P* < 0.05) (Table 7).

**Table 7:** Correlation analysis between fear of progression and MG-ADL score, MGFA classification, STAI score, and TAI score in patients with myasthenia gravis.

Variables	Physiological health dimension	Socio-family dimension	Fear of progression
STAI	−0.003	−0.040	−0.015
TAI	−0.113	−0.085	−0.106
MGFA	0.153	0.235*	0.229*
ADL	0.164	0.174	0.172

Abbreviations: * At level 0.05, the correlation was significant.

To clarify the related variables influencing the FoP in MG patients, the total FoP score was defined as the dependent variable. Statistically significant variables identified through univariate analysis and correlation analysis were selected as independent variables. A multiple linear regression model was constructed to assess their impact on FoP. The assignment of independent variables was as follows: Gender (“male” coded as 1, “female” as 2), MG Progression (“Yes” = 1, “No” = 2), and MGFA classification (Class I = 1, Class II = 2, Class III = 3, Class IV = 4, Class V = 5). The collinearity of independent variables was evaluated using Variance Inflation Factors (VIF), all of which were less than 5 in this study. This indicates that there was no multicollinearity issue among the selected independent variables, ensuring reliable regression analysis results. Using SPSS software for regression modeling, the results demonstrated that gender and MG progression were significant factors influencing FoP in MG patients, collectively accounting for 20.7% of the total variation in the model (Table 8).

**Table 8:** Multi-factor analysis of fear of progression in myasthenia Gravis patients.

Variables	Unstandardized coefficient		Standardized Coefficient			Collinearity Statistics	
	B	Standard error	Beta	T-value	*P*-value	Tolerance	VIF
**(Constant)**	34.374	5.252		6.545	0.000		
**Gender**	6.872	2.018	0.338	3.406	0.001	0.981	1.020
**Progress of MG**	−7.761	2.182	− 0.369	−3.557	0.001	0.896	1.116
**MGFA**	− 0.465	1.318	− 0.037	− 0.353	0.725	0.882	1.134
**Adjusted R** ^**2**^	0.207						
**F**	8.155						
**P**	< 0.001						

*P* < 0.05 was taken as the test standard.

### Comorbidity between FoP and anxiety disorders

**Fig. 1:**
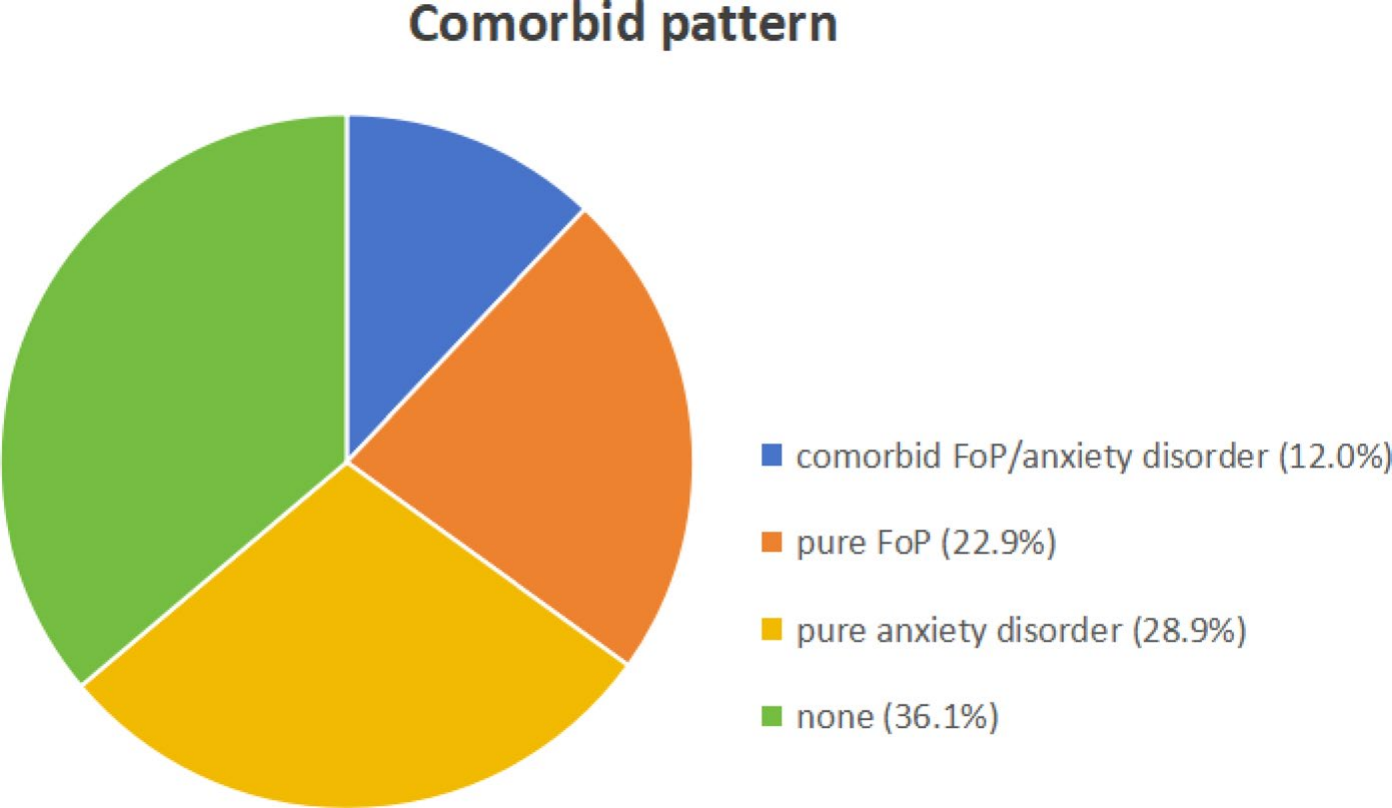
Comorbidity patterns FoP and anxiety disorders.

The results revealed that 36.1% of MG patients exhibited neither clinical FoP nor an anxiety disorder (“none”). Furthermore, 22.9% exhibited clinical-level FoP without an anxiety disorder (“pure FoP”). Additionally, 28.9% were diagnosed with a “pure anxiety disorder” without comorbid clinical FoP. Lastly, 12.0% exhibited comorbid clinical FoP and anxiety disorder (“comorbid FoP/anxiety disorder”) (Fig. 1).

## Discussion

FoP represents a response to both real and perceived threats associated with the diagnosis, treatment, and clinical course of the disease. Among patients, anxiety levels vary widely, ranging from mild discomfort to severe distress^11,14,15,19^. MG is characterized by a chronic progressive course marked by muscle fatigue and weakness, often fluctuating between relapse and remission. The management of MG requires a multidimensional therapeutic approach, as some medications may lead to complications. Due to these factors, including symptom variability and the complexity of therapy regimens, patients may encounter significant challenges in their daily lives, such as work limitations and financial pressures, which can exacerbate psychological distress and induce heightened fear of disease progression^20^. However, excessive FoP can lead to substantial behavioral dysfunction, reduced well-being, and diminished quality of life. Recognizing and addressing this issue among patients with MG is essential.

We conducted an initial assessment of patients’ fear using FoP-Q-SF. In this study, the mean FoP-Q-SF score for MG patients was 31.36, which was significantly lower than the scores of breast cancer patients at 33.84 and acute myocardial infarction patients at 33.43, but higher than that of diabetic patients at 26.84^10,21,22^. The reason for this discrepancy may be that MG is a chronic disease with established treatment plans, allowing patients to manage their condition and delay disease progression through various methods, even though it cannot be fully cured. In contrast, cancer and myocardial infarction patients often face more severe physical and emotional challenges due to the possibility of recurrence or life-threatening complications, which contributes to higher fear levels among these groups. Moreover, 38.6% of MG patients exhibited dysfunctional FoP, a proportion significantly higher than that of diabetic patients at 23.1%^21^. These findings suggest that a considerable number of MG patients experience functional impairments in fear processing. Although both diabetes and MG are chronic conditions requiring long-term medication, the higher proportion of FoP impairment in MG patients may be due to the severe symptoms and complications associated with acute disease progression, such as difficulty breathing and swallowing, which appear to be more frightening than hypoglycemia episodes caused by diabetes. Therefore, medical staff should pay attention to the assessment of FoP in MG patients and implement appropriate measures to alleviate it, such as increasing patient education to help them better understand their condition and improve treatment adherence.

The three items with the highest score were “I’m afraid the drugs will damage my body”, “The thought of being less productive because of illness annoys me” and “The idea that I might not be able to work because of illness bothers me”. However, the findings in our study were different from brain cancer patients, who were more concerned about “what will become of the family if something happens to me” and “severe medical treatments in the course of the illness”^23^. This difference may be related to the characteristics of the disease. The weakness symptoms in MG patients may affect their daily activities and work. Additionally, some steroid or immunotherapy treatments may cause adverse reactions, so patients are concerned about the side effects of medication and the impact of the disease on their work, while cancer patients are more concerned about survival rates and the impact on their families^20^. Therefore, different psychological problems should be addressed for patients with different chronic diseases.

We observed a trend toward that higher Fear of Progression (FoP) scores among patients with lower monthly income (< 3000 yuan/month), those divorced, and those with a history of previous crises. Although the difference did not reach statistical significance, this trend aligns with previous findings suggesting that socioeconomic disadvantage may contribute to psychological distress in chronic illnesses, including MG. Lower-income patients often face greater financial burden due to long-term treatment costs, reduced work capacity, and limited access to healthcare resources^24^. A previous study highlighted the substantial economic and emotional strain experienced by MG patients with lower socioeconomic status^25^, which may exacerbate fears related to disease progression, treatment dependency, and future uncertainty. While our study did not demonstrate a significant association, the observed pattern suggests that economic hardship may be an important contextual factor influencing patients’ psychological adaptation and warrants further investigation in larger cohorts.

Our study demonstrated that gender is significantly associated with FoP levels, with female patients exhibiting higher levels of FoP. A previous prospective cohort study on patients with myasthenia gravis (MG) showed that women exhibited greater disease severity and poorer outcomes across all measured parameters compared to men, which may partially explain the higher FoP levels observed in women^26^. Our findings highlight gender differences in FoP levels among patients with MG. Although the underlying mechanisms remain unclear, these results suggest that clinical practice should take such differences into account. Additionally, the progression of MG history was identified as another critical factor associated with FoP levels. A study reported that relapse occurs in 34% of MG patients^27^, and the intrinsic burden of the disease, compounded by recurrent episodes of myasthenia, likely contributes to elevated FoP levels among MG patients^28^. Our results indicate that MG patients with a history of the disease tend to have higher FoP levels, emphasizing the need for enhanced attention to the assessment and management of such patients in clinical practice. A recent study also found that disease severity in MG patients was associated with depressive symptoms, sleep disturbance, and cognitive impairment^29^. Our findings support this correlation between MG severity and psychological burden, although we focused on FoP rather than cognitive aspects. Additionally, while no significant associations were found between specific comorbidities and FoP in our cohort, the potential impact of neurological conditions such as cerebrovascular disease warrants further investigation in future studies.

FoP is commonly expressed by patients with chronic diseases, and it is understandable that such concerns evoke worry, fear, and anxiety about the future^30^. However, it remains unclear whether MG patients who exhibit clinical FoP are those who, in fact, suffer from a psychiatric anxiety disorder. Is FoP equivalent to an anxiety disorder? To address this question, we conducted two lines of investigation. Firstly, we performed a correlational analysis and found no significant correlation between anxiety scores and FoP scores. The lack of correlation may arise because anxiety encompasses a broader spectrum of emotional responses, while FoP is more focused on concerns regarding the progression of the disease itself. Secondly, we investigated comorbidity patterns between FoP and anxiety disorders in MG patients undergoing treatment. Our findings revealed that there exists a subgroup of MG patients characterized by FoP that does not meet the diagnostic criteria for an anxiety disorder. These patients experience comparable psychological burdens from accompanying mental symptoms as those MG patients who suffer from an anxiety disorder. This discovery aligns with the conclusions of some researchers who emphasize that clinical FoP differs from neurotic anxiety disorders and that core diagnostic criteria for anxiety disorders do not readily apply to FoP^30^. In summary, our findings suggest that FoP in MG patients represents a phenomenon distinct to some extent, with current diagnostic criteria for anxiety disorders failing to fully capture this construct. Therefore, assessing FoP in MG patients is essential for comprehensive clinical care and research.

This study investigated the origins and extent of FoP among MG patients, further elucidating factors related to FoP. However, certain limitations should be acknowledged. (1) Although comorbidities were recorded, we did not conduct a detailed analysis of how different comorbidities (such as cancer or thymoma) might influence the level of FoP. Future studies should include a classification of comorbidities to explore their potential impact. (2) We did not include diagnostic delay or hospitalization data, which may influence patients’ psychological responses. Further research should address these factors. (3) Additionally, we did not include diagnostic delay or hospitalization data, which may influence patients’ psychological responses. Further research should address these factors. 4)This study was conducted among MG patients recruited from a single-center neurology department in Jiangxi Province, China. To enhance the generalizability of the findings, future studies should involve multicenter cohorts and broader geographic regions to identify additional predictors of FoP across diverse patient populations.

## Conclusions

In summary, 38.6% of MG patients developed dysfunction FoP in this study. The presence of FoP among MG patients was influenced by gender and the history of MG progression. No significant correlation was observed between anxiety scores and FoP scores, and the diagnostic criteria for anxiety disorders do not readily apply to FoP. These findings underscore the necessity of assessing FoP in MG patients. Interventions should be tailored to individual patient profiles.

## Electronic supplementary material

Below is the link to the electronic supplementary material.

Supplementary Material 1

## Data Availability

The original contributions presented in the study are included in the article material, further inquiries can be directed to the corresponding authors.

## References

[1] CherukupallyKR, KodjoK, Ogunsakin, O., Olayinka, O. & FouronP Comorbid depressive and anxiety symptoms in a patient with myasthenia Gravis. *Case Rep. Psychiatry*. **2020** (8967818). 10.1155/2020/8967818 (2020). 10.1155/2020/8967818 PMC6984743 32089937

[2] Gilhus, N. E. & Verschuuren, J. J. Myasthenia gravis: Subgroup classification and therapeutic strategies. *Lancet Neurol.* **14**(10), 1023–1036. 10.1016/s1474-4422(15)00145-3 (2015). 26376969 10.1016/S1474-4422(15)00145-3

[3] Berrih-Aknin, S., Frenkian-Cuvelier, M. & Eymard, B. Diagnostic and clinical classification of autoimmune myasthenia gravis. *J. Autoimmun.* 143–148. 10.1016/j.jaut.2014.01.003 (2014). 24530233 10.1016/j.jaut.2014.01.003

[4] Di Stefano, V. et al. Comorbidity in myasthenia gravis: multicentric, hospital-based, and controlled study of 178 Italian patients. *Neurol. Sciences: Official J. Italian Neurol. Soc. Italian Soc. Clin. Neurophysiol.* **45** (7), 3481–3494. 10.1007/s10072-024-07368-0 (2024). 10.1007/s10072-024-07368-0 PMC11176220 38383750

[5] Kulaksizoglu, I. B. Mood and anxiety disorders in patients with myasthenia gravis: Aetiology, diagnosis and treatment. *CNS Drugs* **21**(6), 473–481. 10.2165/00023210-200721060-00004 (2007). 17521227 10.2165/00023210-200721060-00004

[6] Paul, R. H., Cohen, R. A., Goldstein, J. M. & Gilchrist, J. M. Severity of mood, self-evaluative, and vegetative symptoms of depression in myasthenia gravis. *J. Neuropsychiatry Clin. Neurosci.* **12**(4), 499–501. 10.1176/jnp.12.4.499 (2000). 11083168 10.1176/jnp.12.4.499

[7] Qiu, L. et al. [Study of incidence and correlation factors of depression, anxiety and insomnia in patients with myasthenia gravis]. *Zhonghua Yi Xue Za Zhi*. **90** (45), 3176–3179 (2010). 21223762

[8] Yang, Y. et al. Quality of life in 188 patients with myasthenia gravis in China. *Int. J. Neurosci.* **126**(5), 455–462. 10.3109/00207454.2015.1038712 (2016). 26000922 10.3109/00207454.2015.1038712

[9] Dankert, A. et al. [Fear of progression in patients with cancer, diabetes mellitus and chronic arthritis]. *Die Rehabilit.* **42** (3), 155–163. 10.1055/s-2003-40094 (2003). 10.1055/s-2003-40094 12813652

[10] He, J. L. et al. Fear of disease progression among breast cancer patients in China: A meta-analysis of studies using the fear of progression questionnaire short form. *Front. Psychol.* **14**, 1222798. 10.3389/fpsyg.2023.1222798 (2023). 37680239 10.3389/fpsyg.2023.1222798 PMC10482266

[11] Hinz, A., Mehnert, A., Ernst, J., Herschbach, P. & Schulte, T. Fear of progression in patients 6 months after cancer rehabilitation-a- validation study of the fear of progression questionnaire FoP-Q-12. *Support. Care Cancer* **23**(6), 1579–1587. 10.1007/s00520-014-2516-5 (2015). 25412727 10.1007/s00520-014-2516-5

[12] Xiong, M. et al. The impact of fear of cancer recurrence on the quality of life of breast cancer patients: A longitudinal study of the mediation effect of cortisol and hope. *Eur. J. Oncol. Nursing: Official J. Eur. Oncol. Nurs. Soc.* **70**, 102600. 10.1016/j.ejon.2024.102600 (2024). 10.1016/j.ejon.2024.102600 38795441

[13] Zimmermann, T., Herschbach, P., Wessarges, M. & Heinrichs, N. Fear of progression in partners of chronically ill patients. *Behav. Med. (Washington DC)*. **37** (3), 95–104. 10.1080/08964289.2011.605399 (2011). 10.1080/08964289.2011.605399 21895427

[14] Donald, M. et al. Mental health issues decrease diabetes-specific quality of life independent of glycaemic control and complications: Findings from Australia’s living with diabetes cohort study. *Health Qual. Life Outcomes* **11**, 170. 10.1186/1477-7525-11-170 (2013). 24131673 10.1186/1477-7525-11-170 PMC3853250

[15] Grammes, J. et al. Fear of hypoglycemia in patients with type 2 diabetes: The role of interoceptive accuracy and prior episodes of hypoglycemia. *J. Psychosom. Res.* **105**, 58–63. 10.1016/j.jpsychores.2017.12.010 (2018). 29332635 10.1016/j.jpsychores.2017.12.010

[16] Gong, M., Dong, H., Tang, Y., Huang, W. & Lu, F. Effects of aromatherapy on anxiety: A meta-analysis of randomized controlled trials. *J. Affect. Disorders 2020*, **274**:1028–1040. 10.1016/j.jad.2020.05.118 10.1016/j.jad.2020.05.118 32663929

[17] Mehnert, A., Herschbach, P., Berg, P., Henrich, G. & Koch, U. [Fear of progression in breast cancer patients–validation of the short form of the fear of progression questionnaire (FoP-Q-SF)]. *Z. Psychosomat. Med. Psychother.* **52** (3), 274–288. 10.13109/zptm.2006.52.3.274 (2006). 10.13109/zptm.2006.52.3.274 17156600

[18] Herschbach, P. et al. Group psychotherapy of dysfunctional fear of progression in patients with chronic arthritis or cancer. *Psychother. Psychosom.* **79**(1), 31–38. 10.1159/000254903 (2010). 19887889 10.1159/000254903

[19] Indelicato, L. et al. Psychological distress, self-efficacy and glycemic control in type 2 diabetes. *Nutr. Metabolism Cardiovasc. Diseases: NMCD*. **27** (4), 300–306. 10.1016/j.numecd.2017.01.006 (2017). 10.1016/j.numecd.2017.01.006 28274728

[20] Law, C., Flaherty, C. V. & Bandyopadhyay, S. A review of psychiatric comorbidity in myasthenia Gravis. *Cureus* **12** (7), e9184. 10.7759/cureus.9184 (2020). 32802619 10.7759/cureus.9184 PMC7425828

[21] Wang, Y. et al. Predictors of fear of diabetes progression: A multi-center cross-sectional study for patients self-management and healthcare professions education. *Front. Public Health* **10**, 910145. 10.3389/fpubh.2022.910145 (2022). 36600932 10.3389/fpubh.2022.910145 PMC9806215

[22] Wang, X. et al. Fear of progression in patients with acute myocardial infarction: A cross-sectional study. *BMC Nurs.* **23**(1), 866. 10.1186/s12912-024-02552-1 (2024). 39609789 10.1186/s12912-024-02552-1 PMC11606214

[23] Goebel, S. & Mehdorn, H. M. Fear of disease progression in adult ambulatory patients with brain cancer: prevalence and clinical correlates. *Supportive Care Cancer: Official J. Multinational Association Supportive Care Cancer*. **27** (9), 3521–3529. 10.1007/s00520-019-04665-9 (2019). 10.1007/s00520-019-04665-9 30684045

[24] Kankeu, H. T., Saksena, P., Xu, K. & Evans, D. B. The financial burden from non-communicable diseases in low- and middle-income countries: A literature review. *Health Res. Policy Syst.* **11**, 31. 10.1186/1478-4505-11-31 (2013). 23947294 10.1186/1478-4505-11-31 PMC3751656

[25] Lehnerer, S. et al. Burden of disease in myasthenia gravis: Taking the patient’s perspective. *J. Neurol.* **269**(6), 3050–3063. 10.1007/s00415-021-10891-1 (2022). 34800167 10.1007/s00415-021-10891-1 PMC9120127

[26] Gable, K. L. & Hobson-Webb, L. D. What is the significance of gender difference in myasthenia gravis?. *Muscle Nerve* **64**(5), 515–516. 10.1002/mus.27397 (2021). 34383342 10.1002/mus.27397

[27] Wang, L., Zhang, Y. & He, M. Clinical predictors for the prognosis of myasthenia gravis. *BMC Neurol.* **17**(1), 77. 10.1186/s12883-017-0857-7 (2017). 28420327 10.1186/s12883-017-0857-7 PMC5395963

[28] Nelke, C. et al. Independent risk factors for myasthenic crisis and disease exacerbation in a retrospective cohort of myasthenia Gravis patients. *J. Neuroinflamm.* **19** (1), 89. 10.1186/s12974-022-02448-4 (2022). 10.1186/s12974-022-02448-4 PMC9005160 35413850

[29] Iacono, S. et al. Frequency and correlates of mild cognitive impairment in myasthenia Gravis. *Brain Sci.* **13** (2). 10.3390/brainsci13020170 (2023). 10.3390/brainsci13020170 PMC9953757 36831713

[30] Dinkel, A., Kremsreiter, K., Marten-Mittag, B. & Lahmann, C. Comorbidity of fear of progression and anxiety disorders in cancer patients. *Gen. Hosp. Psychiatry*. **36** (6), 613–619. 10.1016/j.genhosppsych.2014.08.006 (2014). 25213227 10.1016/j.genhosppsych.2014.08.006

