# Supplementary material for: A cross-sectional study on fear of progression in patients with myasthenia gravis
Source: Sci Rep. 2025 Jul 18;15:26181. doi: 10.1038/s41598-025-11806-0 (PMC12274404; doi:10.1038/s41598-025-11806-0)
Supplement: Supplementary file 1 — Supplementary Material 1 [file 41598_2025_11806_MOESM1_ESM.docx]

| **Diagnostic method** | **Number of patients (n)** | **Percentage (%)** |
| --- | --- | --- |
| Positive AChR antibody | 83 | 100.00% |
| Abnormal RNS | 22 | 26.50% |
| Positive neostigmine test | 33 | 39.76% |
| Diagnosis supported by ≥2 modalities | 51 | 61.45% |

**Supplement Table 1. Diagnosis-related data of the included MG patients.**

**Supplement Table 2. Specific types and numbers of comorbidities in patients.**

| **Comorbidity** | **Number of patients (n)** | **Percentage (%)** |
| --- | --- | --- |
| Hypertension | 12 | 14.46% |
| Diabetes | 10 | 12.05% |
| Hyroid dysfunction | 8 | 9.64% |
| Kidney disease | 6 | 7.23% |
| Liver disease | 3 | 3.61% |
| Osteoporosis | 3 | 3.61% |
| Cerebrovascular disease | 1 | 1.20% |
| Dementia | 1 | 1.20% |
